# Status of Systemic Oxidative Stresses in Patients with Primary Open-Angle Glaucoma and Pseudoexfoliation Syndrome

**DOI:** 10.1371/journal.pone.0049680

**Published:** 2012-11-26

**Authors:** Masaki Tanito, Sachiko Kaidzu, Yasuyuki Takai, Akihiro Ohira

**Affiliations:** Department of Ophthalmology, Shimane University Faculty of Medicine, Izumo, Shimane, Japan; Baker IDI Heart and Diabetes Institute, Australia

## Abstract

**Background:**

The involvement of local and systemic oxidative stress in intraocular pressure (IOP) elevation and optic nerve damage has been hypothesized in the pathogenesis of glaucoma. To test this, we measured the systemic levels of prooxidants and antioxidants by analyzing the blood biochemistry in patients with glaucoma.

**Methods:**

Peripheral blood samples were collected from Japanese patients with primary open-angle glaucoma (PG) (n = 206), exfoliation syndrome (EX) (n = 199), and controls (n = 126). Plasma levels of lipid peroxides, ferric-reducing activity, and thiol antioxidant activity were measured by diacron reactive oxygen metabolites (dROM), biological antioxidant potential (BAP), and sulfhydryl (SH) tests, respectively, using a free radical analyzer.

**Results:**

In the PG, EX, and control groups, the mean ± standard deviation values were 355±63, 357±69, and 348±56 (U. Carr), respectively, for dROM; 1,951±282, 1,969±252, and 2,033±252 (µmol/L), respectively, for BAP (µmol/L); and 614±98, 584±91, and 617±99 (µmol/L), respectively, for SH. The differences in the BAP values were significant between the PG and control groups (p = 0.0062), for SH between the EX and control groups (p = 0.0017), and for SH between the PG and EX groups (p = 0.0026). After adjustment for differences in age and sex among groups using multiple regression analysis, lower BAP values were correlated significantly with PG (p = 0.0155) and EX (p = 0.0049). Higher dROM values with and without glaucoma were correlated with female gender, and lower SH values with older age. There were no significant differences between the higher (≥21 mmHg) and lower (<21 mmHg) baseline IOPs in the PG group or between the presence or absence of glaucoma in the EX group.

**Conclusions:**

Lower systemic antioxidant capacity that measured by ferric-reducing activity is involved in the pathogenesis of PG and EX.

## Introduction

Glaucoma, characterized by progressive “glaucomatous” optic neuropathy and corresponding visual field loss, is a leading cause of irreversible blindness worldwide [1], [2], including in Japan [3]. Retinal ganglion cell (RGC) death due to apoptosis and loss of RGC axons leads to glaucomatous optic neuropathy, in which elevated intraocular pressure (IOP) is the main risk factor [2]. In open-angle glaucoma (OAG) including primary open-angle glaucoma (PG) and glaucoma secondary to pseudo-exfoliation syndrome (EX), the IOP increases because of reduced aqueous humor outflow at the trabecular meshwork (TM) [4]. The increased aqueous humor outflow resistance results from changes in the amount and quality of the extracellular matrix (ECM) in the TM [5]. In EX, an age-related, complex, generalized disorder of the ECM, the progressive accumulation of intraocular abnormal fibrillar materials in the TM is considered the primary cause of chronic IOP elevation [6], [7]. Although the involvement of various genetic and internal and external stress factors, such as immune reactions [8], [9], inflammation [10], ischemia [11], and hypoxia [12], have been proposed, the exact mechanisms of the ECM changes in the TM and progressive loss of RGC bodies and axons in OAG is still not well understood. Clinical and experimental studies have shown the possible involvement of oxidative stress in the pathogenesis of glaucoma, including increased aqueous outflow resistance with increased dysfunction of the TM cell viability [13]–[15] and glaucomatous neurodegeneration in the retina, optic nerve, and brain [16], [17]. Thus, oxidative stress seems to explain the wide range of tissue and/or cellular damage in glaucoma.

Oxidative stress generally is induced through formation of multiple reactive oxygen species including superoxide, hydrogen peroxide, and hydroxyl radicals that can initiate and propagate free radicals. The net oxidative burden between prooxidant and antioxidant systems is the oxidative stress that damages cellular and tissue macromolecules such as lipids, proteins, and nucleic acids, and results in cellular and tissue dysfunction and cellular death. After identification of the circulating autoantibodies against antioxidative stress enzymes and chaperone molecules glutathione S-transferase [18] and heat shock proteins [19], [20] in the serum of patients with glaucoma, the systemic status of redox (reduction/oxidation) in glaucoma has become a topic of interest. Until now, a number of studies have assessed this topic [21]–[29]. However, these studies may not be sufficiently comprehensive in that they included relatively small numbers of subjects (mostly only 20–40 subjects in each case and control groups), analyzed only one glaucoma type (either PG or EX) or unclassified mixed types of glaucoma in the case group, and/or analyzed either prooxidant or antioxidant status only.

Therefore, the aim of the current study was to examine the systemic redox status of glaucoma more comprehensively by including more patients with PG and EX in each group and by simultaneously testing the oxidative and antioxidative status.

## Subjects and Methods

### Subjects

A total of 531 Japanese subjects comprised the PG (n = 206), EX (n = 199), and non-glaucomatous control (n = 126) groups. These subjects were recruited consecutively at Shimane University Hospital and Iinan Hospital, Shimane, Japan. The current study adhered to the tenets of the Declaration of Helsinki. The institutional review boards of both hospitals reviewed and approved the research. All subjects provided written informed consent. All subjects underwent ophthalmologic examinations including measurements of the best-corrected visual acuity (VA) and IOP by Goldmann applanation tonometry and slit-lamp, gonioscopic, and funduscopic examinations under pupillary dilatation. PG was diagnosed based on the presence of an open iridocorneal angle bilaterally, the characteristic appearance of glaucomatous optic neuropathy such as enlargement of the optic disc cup or focal thinning of the neuroretinal rim, and corresponding visual field defects identified using the Humphrey Visual Field Analyzer (Carl Zeiss Meditec, Dublin, CA) in at least one eye, and no evidence of secondary glaucoma bilaterally. In the PG group, 117 subjects with the history of untreated IOP of 21 mmHg or higher in at least one eye were considered to have high-tension glaucoma (HTG), and the other 89 subjects who did not have a history of untreated IOP of 21 mmHg or higher were considered to have normal tension glaucoma (NTG). EX was diagnosed based on the presence of an open iridocorneal angle and characteristic pseudoexfoliation material deposits on the anterior capsule and/or pupillary margin in at least in one eye. In the EX group, 131 subjects with a history of IOP elevations of 21 mmHg or higher or the characteristic appearance of glaucomatous optic neuropathy in at least in one eye were considered to be exfoliation glaucoma (EXG), and the other 68 subjects who did not have a history of IOP elevations and glaucomatous optic neuropathy were considered to be exfoliation syndrome without glaucoma (EXS). The control subjects had a corrected visual acuity (VA) of 0.7 or better measured in both eyes using a decimal VA chart, and no glaucomatous optic neuropathy or history of IOP of 21 mmHg or higher. Except for a cataract and/or glaucoma, no subjects had ocular pathologies such as clinically detectable ocular inflammation, infection, neuropathies, retinopathies, or maculopathies.

### Recording Clinical Parameters and Collecting Blood Samples

To avoid the possible confounding effect of systemic diseases [30]–[32], the subjects were questioned about a history of severe systemic diseases during an interview before entry into the study. These diseases included acute brain infarction and hemorrhage, systemic neurologic diseases, cardiac diseases requiring catheter placement or surgery, cardiac failure and other systemic diseases affecting the subjects' physical activity, lung diseases causing dyspnea, chronic and acute hepatitis requiring interferon therapy, liver cirrhosis, renal failure requiring hemodialysis, autoimmune diseases requiring systemic steroids and other immunosuppressive therapies, severe anemia requiring blood transfusions, major visceral surgery, malignancies, and severe hypertension causing cardiac and kidney failure, severe diabetes requiring insulin therapy. In addition to a history of severe systemic diseases, to adjust for the possible confounding effects of other factors such as differences in nutrition, blood pressure, blood glucose, and smoking habits [33]–[35], the presence or absence of diabetes, current smoking, the time since the last meal, and the systolic (SBP) and diastolic (DBP) blood pressures and pulse rate were recorded before the blood sample were collected. Venous blood specimens were collected from the antecubital vein into evacuated tubes. Plasma samples obtained by centrifugation of the collected venous blood were stored at 4°C until oxidative stress measurements. During all handling procedures, including transportation from the clinical setting to the laboratory and centrifugation, the temperature was maintained at 4°C.

### Oxidative Stress Measurements

All blood analyses were performed using a free radical analyzer system (FREE Carpe Diem, Wimerll Company Ltd., Tokyo, Japan) that included a spectrophotometric device reader and a thermostatically regulated mini-centrifuge, and the measurement kits were optimized to the FREE Carpe Diem System, according to the manufacturer's instructions. Based on the recommendation from the manufacturer, all analyses were performed within 48 hours of venous blood collection to avoid falsely high or low results. To analyze the plasma levels of reactive oxygen metabolites, antioxidant capacity, and thiol-antioxidant capacity, diacron reactive oxygen metabolite (dROM), biological antioxidant potential (BAP), and sulfhydryl (SH) tests were performed, respectively.

The dROM test reflects the amount of organic hydroperoxides that is related to the free radicals from which they are formed. When the samples are dissolved in an acidic buffer, the hydroperoxides react with the transition metal (mainly iron) ions liberated from the proteins in the acidic medium and are converted to alkoxy and peroxy radicals. These newly formed radicals oxidize an additive aromatic amine (*N,N*-diethyl-*para*-phenylen-diamine) and cause formation of a relatively stable colored cation radical that is spectrophotometrically detectable at 505 nm [33], [36]. The results are expressed in arbitrary units (U. Carr), one unit of which corresponds to 0.8 mg/L of hydrogen peroxide [33], [36].

The BAP test provides an estimate of the global antioxidant capacity of blood plasma, measured as its reducing potential against ferric ions. When the sample is added to the colored solution obtained by mixing a ferric chloride solution with a thiocyanate derivative solution, decoloration results. The intensity of the decoloration is spectrophotometrically detectable at 505 nm and is proportional to the ability of plasma to reduce ferric ions [34], [37]. The results are expressed in µmol/L of the reduced ferric ions.

The SH test provides an estimate of the total thiol groups in the biologic samples, using a modified Ellman method [38], [39]. When the sample is added to the solution, sulfhydryl groups in the sample react with 5,5-dithiobus-2-nitrobenzoic acid, which is followed by development of a stained complex that is spectrophotometrically detectable at 405 nm and is proportional to their concentration according to the Beer-Lambert law [34], [36]. The results are expressed as µmol/L of the sulfhydryl groups.

### Statistical Analysis

Data are expressed as the mean ± standard deviation and analyzed using StatView version 5.01 statistical software (SAS Institute, Inc., Cary, NC). For comparisons between the control, PG, and EX groups, the differences in continuous data (age, SBP, DBP, pulse rate, time from the last meal, dROM, BAP, and SH tests) were calculated using one-way analysis of variance followed by post-hoc un-paired t tests, and the differences in categorical data (sex, diabetes, and current smoking) were calculated using the G-test followed by the post-hoc Fisher's exact probability test. To correct for multi-group comparisons, p values of 0.0167 and 0.0033 for the unpaired or Fisher's exact probability test were considered significant with significance levels of 5% and 1%, respectively, based on the Bonferroni's method. The same statistical tests were used for the comparisons among the control, NTG, and HTG groups or among the control, EXS, and EXG groups. To adjust for differences in age and sex distributions among the groups, the values of the dROM, BAP, and SH tests were compared between the PG and control groups or the EX and control groups using multiple regression analysis; p values of 0.0167 and 0.0033 were considered significant with significance levels of 5% and 1%, respectively.

## Results

The demographic patient data, including age, sex, SBP, DBP, pulse rate, time from the last meal, diabetes, and smoking status, are summarized in Table 1. The comparisons among the control, PG, and EX groups showed that the patients in the EX group were significantly older (78.2 years) than those in the control (70.6 years, p<0.0001) and the PG (70.5 years, p<0.0001) groups, and there were significantly fewer women in the PG (53.9%, p = 0.0113) and EX (51.8%, p = 0.0039) groups than in the control group (68.3%). Other parameters did not differ significantly among the three groups. The subgroup analyses among the controls (68.3%), NTG, and HTG groups showed that significantly fewer women had HTG (49.6%, p = 0.0040) compared with the control group, and the DBP was significantly higher in the HTG (78.7 mmHg) group than in the control (74.6 mmHg, p = 0.0132) and the NTG (74.2 mmHg, p = 0.0133) groups. The subgroup analyses among the control, EXS, and EXG groups showed that significantly fewer women had EXG (41.2%) compared with the control (68.3%, p<0.0001) and EXS (72.1%, p<0.0001) groups.

**Table 1:** Demographic subject data.

	Control	PG			EX			p value
		Total	NTG	HTG	Total	EXS	EXG	
N	126	206	89	117	199	68	131	
Age (yrs)								
Mean ± SD	70.6±10.8	70.5±11.3	72.6±10.7	68.9±11.5	78.2±7.8	78.6±7.3	78.0±8.1	<0.0001^a^**
range	23–93	24–89	25–89	24–88	50–96	61–96	50–94	
p value, v.s. CT ^c^		0.9677	0.1694	0.2534	<0.0001##	<0.0001##	<0.0001##	
p value, v.s. PG^ c^		-	-	-	<0.0001##	-	-	
p value, v.s. NTG^ c^		-	-	0.0196	-	-	-	
p value, v.s. EXS^ c^		-	-	-	-	-	0.5852	
Sex								
Men, n (%)	40 (31.7)	95 (46.1)	36 (40.4)	59 (50.4)	96 (48.2)	19 (27.9)	77 (58.8)	0.0079^b^**
Women, n (%)	86 (68.3)	111 (53.9)	53 (59.6)	58 (49.6)	103 (51.8)	49 (72.1)	54 (41.2)	
p value, v.s. CT^ d^		0.0113#	0.1960	0.0040#	0.0039#	0.6265	<0.0001##	
p value, v.s. PG^ d^		-	-	-	0.6912	-	-	
p value, v.s. NTG^ d^		-	-	0.1616	-	-	-	
p value, v.s. EXS^ d^		-	-	-	-	-	<0.0001##	
SBP (mmHg)								
Mean ± SD	136.2±19.4	139.8±20.0	139.7±19.4	139.9±20.5	141.1±21.1	139.2±21.1	142.1±21.1	0.0964^a^
range	94–223	89–211	90–182	89–211	85–203	85–203	85–191	
p value, v.s. CT ^c^		0.1146	0.1951	0.1462	0.0326	0.3156	0.0197	
p value, v.s. PG^ c^		-	-	-	0.5124	-	-	
p value, v.s. NTG^ c^		-	-	0.9829	-	-	-	
p value, v.s. EXS^ c^		-	-	-	-	-	0.3563	
DBP (mmHg)								
Mean ± SD	74.6±12.0	76.8±12.9	74.2±11.3	78.7±13.7	76.1±13.2	73.2±10.7	77.7±14.2	0.3223^a^
range	45–119	47–133	47–100	51–133	46–123	51–100	46–123	
p value, v.s. CT ^c^		0.1349	0.8182	0.0132#	0.2929	0.4167	0.0626	
p value, v.s. PG^ c^		-	-	-	0.6189	-	-	
p value, v.s. NTG^ c^		-	-	0.0133#	-	-	-	
p value, v.s. EXS^ c^		-	-	-	-	-	0.0237	
Pulse rate (/min.)								
Mean ± SD	73.3±12.0	75.3±14.6	73.3±14.2	76.8±14.7	75.5±12.6	73.4±12.2	76.6±12.7	0.2914^a^
range	46–109	42–131	51–114	42–131	48–120	48–120	53–112	
p value, v.s. CT ^c^		0.1817	0.9842	0.0425	0.1407	0.9581	0.0315	
p value, v.s. PG^ c^		-	-	-	0.8663	-	-	
p value, v.s. NTG^ c^		-	-	0.0906	-	-	-	
p value, v.s. EXS^ c^		-	-	-	-	-	0.0847	
Duration from last meal (h)								
Mean ± SD	4.0±2.9	3.8±2.0	3.7±1.3	3.9±2.4	3.8±2.0	3.5±1.3	3.9±1.6	0.6024^a^
range	1–18	1–19	1.5–8	1–19	0.5–14	1.5–7.5	0.5–14	
p value, v.s. CT ^c^		0.3873	0.3084	0.7525	0.3479	0.1879	0.7586	
p value, v.s. PG^ c^		-	-	-	0.9271	-	-	
p value, v.s. NTG^ c^		-	-	0.4279	-	-	-	
p value, v.s. EXS^ c^		-	-	-	-	-	0.0826	
Diabetes								
Yes, n (%)	18 (14.3)	47 (22.8)	15 (16.9)	32 (27.3)	35 (17.6)	13 (19.1)	22 (16.8)	0.1310^b^
No, n (%)	108 (85.7)	159 (77.2)	74 (83.1)	85 (72.7)	164 (82.4)	55 (80.9)	109 (83.2)	
p value, v.s. CT^ d^		0.0644	0.7015	0.0167	0.5379	0.4147	0.6093	
p value, v.s. PG^ d^		-	-	-	0.2167	-	-	
p value, v.s. NTG^ d^		-	-	0.0938	-	-	-	
p value, v.s. EXS^ d^		-	-	-	-	-	0.6978	
Current smoking habit								
Yes, n (%)	12 (9.5)	25 (12.2)	7 (7.9)	18 (15.5)	11 (5.5)	2 (2.9)	9 (6.9)	0.0573^b^
No, n (%)	114 (90.5)	180 (87.8)	82 (92.1)	98 (84.5)	188 (94.5)	66 (97.1)	122 (93.1)	
p value, v.s. CT^ d^		0.4795	0.8089	0.1756	0.1876	0.1440	0.4990	
p value, v.s. PG^ d^		-	-	-	0.0226	-	-	
p value, v.s. NTG^ d^		-	-	0.1313	-	-	-	
p value, v.s. EXS^ d^		-	-	-	-	-	0.3377	

The measured levels of oxidative and antioxidative parameters in plasma of subjects are shown in Table 2. The dROM levels, a measure of oxidative status, did not differ significantly in any comparisons. The BAP levels, a measure of antioxidative status, were significantly lower in the PG group (1,951 µmol/L, p = 0.0062) than the control group (2,033 µmol/L); although the BAP level was lower in the EX (1,969 µmol/L) group than the control group but did not reach significance (p = 0.0360). The subgroup analyses showed that compared to the control group, The BAP level was significantly lower in the HTG group (1,917 µmol/L, p = 0.0005). The SH level, a measure of thiol-mediated antioxidative status, was significantly lower in the EX group (584 µmol/L) than in the control (617 µmol/L, p = 0.0026) and PG (614 µmol/L, p = 0.0017) groups. The subgroup analyses also showed that compared to the control group the SH levels were lower in the EXS (585 µmol/L, p = 0.0227) and EXG (584 µmol/L, p = 0.0066) groups, but the difference did not reach significance between the two (p = 0.9615).

**Table 2:** The dROM, BAP, and SH values.

	Control	PG			EX			*p-value*
		Total	NTG	HTG	Total	EXS	EXG	
n	126	206	89	117	199	68	131	
dROMs test (U. CARR)								
Mean ± SD	347.9±56.0	354.9±62.7	350.7±66.1	358.2±60.1	357.2±69.0	353.3±64.2	359.2±71.5	*0.4280^a^*
range	219–511	204–555	204–555	219–522	102–551	201–501	102–551	
* p-value,* *v.s. CT^b^*		*0.3307*	*0.7425*	*0.1698*	*0.2001*	*0.5428*	*0.1600*	
* p-value,* *v.s. PG^b^*		*-*	*-*	*-*	*0.7177*	*-*	*-*	
* p-value,* *v.s. NTG^b^*		*-*	*-*	*0.3964*	*-*	*-*	*-*	
* p-value,* *v.s. EXS^b^*		*-*	*-*	*-*	*-*	*-*	*0.5681*	
BAP test (µmol/L)								
Mean ± SD	2032.6±251.5	1950.5±282.2	1994.6±301.3	1916.9±263.2	1969.4±252.3	1978.2±301.6	1964.8±223.6	0.0206^a^*
range	1218–2813	990–2857	1242–2857	990–2723	413–2560	413–2492	1355–2560	
* p-value,* *v.s. CT^b^*		*0.0062#*	*0.3162*	*0.0005##*	*0.0360*	*0.1820*	*0.0231*	
* p-value,* *v.s. PG^b^*		*-*	*-*	*-*	*0.4717*	*-*	*-*	
* p-value,* *v.s. NTG^b^*		*-*	*-*	*0.0501*	*-*	*-*	*-*	
* p-value,* *v.s. EXS^b^*		*-*	*-*	*-*	*-*	*-*	*0.7245*	
SH test (µmol/L)								
Mean ± SD	617.0±99.2	614.2±97.9	611.9±97.9	615.9±98.3	584.1±90.7	584.6±83.1	583.9±94.7	0.0014^a^**
range	320–830	328–884	331–884	328–847	257–833	395–760	257–833	
* p-value,* *v.s. CT^b^*		*0.7915*	*0.7068*	*0.9298*	*0.0026##*	*0.0227*	*0.0066#*	
* p-value,* *v.s. PG^b^*		*-*	*-*	*-*	*0.0017##*	*-*	*-*	
* p-value,* *v.s. NTG^b^*		*-*	*-*	*0.7706*	*-*	*-*	*-*	
* p-value,* *v.s. EXS^b^*		*-*	*-*	*-*	*-*	*-*	*0.9615*	

Since the age and sex differed among the control, PG, and EX groups (Table 1), multiple regression analyses were performed to adjust for any possible confounding effects of these two parameters on the measured oxidative and antioxidative status (Table 3). Comparisons between the control and PG groups (Table 3) showed that glaucoma was associated significantly with lower BAP levels (p = 0.0155), while glaucoma was unrelated to the results of the dROM (p = 0.1549) and SH (p = 0.8187) tests. We also found a significant correlation between female gender and higher dROM levels (p = 0.0056) and between older age and lower SH levels (p<0.0001). Comparisons between the control and EX groups (Table 3) showed that EX was related significantly to lower BAP levels (p = 0.0049), while glaucoma was not related to the results of the dROM (p = 0.1938) and SH (p = 0.9488) tests. We also found a significant correlation between female gender and higher dROM levels (p = 0.0007) and between older age and lower SH levels (p<0.0001).

**Table 3:** Multiple regression analyses for dROM, BAP, and SH values.

	PG vs Control (n = 332)			EX vs Control (n = 325)		
	dROM test (U. CARR)	BAP test (µmol/L)	SH test (µmol/L)	dROM test (U. CARR)	BAP test (µmol/L)	SH test (µmol/L)
*R^2^*	0.026	0.038	0.134	0.044	0.033	0.177
*P-value for fitting*	0.0317	0.0050	<0.0001	0.0023	0.0131	<0.0001
Age (per yr)						
* Slope*	−0.02	2.1	−3.3	0.4	3.6	−4.1
* p-value*	0.9564	0.1135	<0.0001**	0.2992	0.0184	<0.0001**
Sex (men = 0, women = 1)						
* Slope*	18.9	49.2	4.1	24.6	13.7	7.3
* p-value*	0.0056*	0.1089	0.6954	0.0007**	0.6331	0.4611
Glaucoma (Control = 0, PG or EX = 1)						
* Slope*	9.7	−75.0	−2.4	10.3	−88.7	−0.7
* p-value*	0.1549	0.0155*	0.8178	0.1938	0.0049*	0.9488

## Discussion

Several studies have assessed the systemic status of redox in patients with glaucoma [21]–[29]. Engin et al. analyzed 160 patients with glaucoma and 31 control subjects in the largest study; however, the study included mixed types of glaucoma in a case group [26]. The various types of glaucoma, i.e., open-angle and angle-closure glaucomas or primary and secondary glaucomas, have different etiologies; thus, separate analyses of each glaucoma type seemed more appropriate. Except for the study by Engin et al. [26], the other studies included up to 40 subjects in the glaucoma groups. The current study of 531 subjects is the largest assessment of the systemic redox status in OAG.

We found that the BAP level, a measure of total antioxidative stress activity, was lower in plasma in the PG and EX groups compared with the control groups after adjustment for age and sex (Table 3). Several studies have reported lower systemic levels of antioxidants or antioxidative stress capacity in glaucoma, that is, a reduced form of the glutathione level was lower in the red blood cells of patients with PG [22], Erel's total antioxidant capacity [40] was lower in the serum of patients with OAG [28], [29] or EX [24], [29], the vitamin E level was lower in the serum of patients with glaucoma [26], and the level of the reduced form of glutathione and the enzymatic activities of catalase, superoxide dismutase, and glutathione peroxidase were lower in the red blood cells of patients with PG [22], [27]. Sorkhabi et al. reported a correlation between a higher aqueous humor 8-hydroxy-2′-deoxyguanosine (8-OHdG) level, an established marker for oxidative stress-induced DNA damage, and a higher 8-OHdG level and lower antioxidant capacity levels in the serum of patients with glaucoma [28]. Thus, the systemic antioxidant capacity could reflect the local ocular redox status. In experimental studies, free-radical scavengers effectively prevented glaucomatous tissue injury such as glutamate- and IOP-induced RGC death [41], [42] and tumor necrosis factor α-induced axonal injury [43]. Collectively, decreased levels of systemic antioxidant capacity might be involved in glaucomatous TM and neuronal damage as a result of local inadequate defense against oxidative stress. Since the decrease in BAP was more marked in HTG and EXG than in NTG and EXS, reduced antioxidant capacity is related more closely to the IOP elevation than neuronal damage. Engin et al. [26] has suggested the possible association between lower systemic antioxidants levels and clinical parameters of glaucoma, thus the analyses regarding the correlations between BAP values and severity indices of glaucoma such as visual field, optic disc morphology, and IOP level using the data obtained in this study would be interest, and should be tested in the near future.

We found no significant difference in the levels of dROM between the control and any glaucoma groups. Several previous studies have reported higher systemic levels of oxidation in glaucoma; the lipid peroxidation byproduct malondialdehyde level is higher in plasma from patients with PG [21], [29], and Erel's total oxidant status measured by the oxidizing activity of the ferrous ion/*o*-dianisidine complex to ferrous ion [44] is higher in the serum from patients with glaucoma [26], PG [29] or EX [24], [29]. In the current study, we included eyes with mild cataract with a VA of 0.7 or better in the control group, while most previous studies have included subjects without cataracts as controls. The presence of a cataract might be related to increased systemic oxidation [45], [46]; thus, the difference in the control groups between the current and previous studies might explain the discrepancy. In addition, the relatively small difference (around 5%) in BAP between the glaucoma and control groups in the current study might have increased if we had included only non-cataractous control eyes. Current study included both newly and previously diagnosed glaucoma patients. One may imagine that the patients who previously diagnosed had already changed their lifestyle to reduce the oxidative stress or increase the anti-oxidative defense. Therefore, in the same context, the difference in BAP between the glaucoma and control groups in the current study might have increased if we had included only newly diagnosed glaucoma eyes. Alternatively, if the absence of a difference in the systemic dROM levels between the glaucoma and control groups is real, glaucomatous tissue damage might result from a local increase in oxidative stress due to local compensation for systemic oxidative stress by systemic reduction of antioxidant capacity in each individual.

The results suggested that with or without glaucoma the dROM level was higher in women than in men, and the SH level, a measure of the total amount of thiol, was correlated negatively with age (Table 3). The results corroborated previous observations regarding a gender difference in systemic oxidative stress [47], [48]. Thus, the evidence supports the suitability of the measurement protocol and statistical analyses in the current study. The glutathione and thioredoxin systems are thought to be major thiol-mediated redox systems in humans, although the plasma glutathione level is 100 to 1,000 times higher than that of thioredoxin [49], [50]. Gherghel et al. reported a negative correlation between age and total glutathione levels in red blood cells with no significant difference in the total glutathione level between the patients with glaucoma and controls [22]. Thus, the SH level in the current study might correspond mainly to the total glutathione level, but that remains to be clarified. Previous studies have reported the possible involvement of thioredoxin system dysregulation in the pathogenesis of glaucoma [41], [42], [51]–[53]; thus, measurement of the thioredoxin level separately from the other thiol groups should be done in the future.

Based on the personally provided information from the manufacturer, that keeping the blood samples at 4°C enables us to obtain a stable acquisition of data for up to 48 hours, we decided to perform measurements within 48 hours from the blood collection. It is generally recognized as that pro- and anti-oxidants are sometimes unstable, thus relatively long duration between the blood collection and the measurements might affect the results. Instead of fasting before the blood collection, we decided to record the duration after the last meal in this study. Although the duration was not different among the study groups, this factor might affect the results also. Use of antioxidants and other supplements were not defined as the inclusion/exclusion criteria of this study, thus this is another weak point of this study.

Based on the results of this comprehensive large-scale study, we concluded that patients with glaucoma have low levels of serum antioxidative capacity, suggesting a general compromise of the antioxidant defense.
