# Correction: Status of Systemic Oxidative Stresses in Patients with Primary Open-Angle Glaucoma and Pseudoexfoliation Syndrome

**DOI:** 10.1371/annotation/35388eb8-2189-4c49-9f45-35cb0911e94c

**Published:** 2014-01-02

**Authors:** Masaki Tanito, Sachiko Kaidzu, Yasuyuki Takai, Akihiro Ohira

The authors measured the results of three oxidative stress markers in blood serum, not plasma. In the second sentence of the "Methods" section of the Abstract, "Plasma levels" should be "Serum levels." In the penultimate sentence of the last paragraph of the "Recording Clinical Parameters and Collecting Blood Samples" section of the "Subjects and Methods", "Plasma samples" should be "Serum samples." In the last sentence of the first paragraph of the "Oxidative Stress Measurements" section of the "Subjects and Methods", "plasma levels" should be "serum levels." In the first sentence of the third paragraph of that same section, "blood plasma" should be "blood." In the first sentence of the second paragraph of the "Results", "plasma" should be "serum." Lastly, in the first sentence of the second paragraph of the "Discussion", "plasma" should be "serum." Since the measurement system the authors used is compatible with both plasma and serum, any data presented and conclusions described in the article are not affected by the correction.

